# Cholesterol Pathways Affected by Small Molecules That Decrease Sterol Levels in Niemann-Pick Type C Mutant Cells

**DOI:** 10.1371/journal.pone.0012788

**Published:** 2010-09-21

**Authors:** Madalina Rujoi, Nina H. Pipalia, Frederick R. Maxfield

**Affiliations:** Department of Biochemistry, Weill Cornell Medical College, New York, New York, United States of America; Thomas Jefferson University, United States of America

## Abstract

**Background:**

Niemann-Pick type C (NPC) disease is a genetically inherited multi-lipid storage disorder with impaired efflux of cholesterol from lysosomal storage organelles.

**Methodology/Principal Findings:**

The effect of screen-selected cholesterol lowering compounds on the major sterol pathways was studied in CT60 mutant CHO cells lacking NPC1 protein. Each of the selected chemicals decreases cholesterol in the lysosomal storage organelles of NPC1 mutant cells through one or more of the following mechanisms: increased cholesterol efflux from the cell, decreased uptake of low-density lipoproteins, and/or increased levels of cholesteryl esters. Several chemicals promote efflux of cholesterol to extracellular acceptors in both non-NPC and NPC1 mutant cells. The uptake of low-density lipoprotein-derived cholesterol is inhibited by some of the studied compounds.

**Conclusions/Significance:**

Results herein provide the information for prioritized further studies in identifying molecular targets of the chemicals. This approach proved successful in the identification of seven chemicals as novel inhibitors of lysosomal acid lipase (Rosenbaum et al, Biochim. Biophys. Acta. 2009, 1791:1155–1165).

## Introduction

Niemann-Pick type C (NPC) disease is a fatal, neurodegenerative disorder associated with an abnormal accumulation of unesterified cholesterol and other lipids (such as sphingomyelin, bis-(monoacylglycerol)-phosphate, glycosphingolipids, and phospholipids) in late endosome/lysosome (LE/LY)-like storage organelles (LSOs) [1], [2], [3], [4], [5], [6], [7]. A defective gene responsible for most cases of NPC disease was identified in 1997 as the *NPC1* gene located on chromosome 18q11 [8]. Later it was determined that about 5% of NPC cases are caused by mutations in another gene, *NPC2*, located on the chromosome 14q24.3 [9]. NPC1 protein is an integral membrane protein associated primarily with late endosomes [10], [11], [12]. NPC2 protein is a cholesterol-binding soluble protein located within the lumen of the LE/LY [9], [13]. The exact roles of these two proteins in the movement of cholesterol out from the LE/LY had not been deciphered [14], [15], [16], [17], [18], [19].

Following the discovery of the link between NPC disease and cholesterol [20], extensive studies were pursued on CHO cells exhibiting an NPC phenotype and on human NPC mutant fibroblasts [2], [3], [8], [15], [21], [22], [23]. This work led to better understanding of the changes in cholesterol transport and metabolism in NPC-deficient cells.

Cholesterol (mostly esterified) is internalized into cells via endocytosis of low-density lipoproteins (LDLs) taken up by the LDL receptors and delivered to LE/LY, where hydrolysis of cholesteryl esters by lysosomal acid lipase (LAL) takes place [24], [25]. In normal cells, the free cholesterol is then redistributed to other organelles, such as the plasma membrane from which the sterol can be effluxed to extracellular acceptors by ATP-binding cassette transporters [26]. LDL-derived cholesterol is also transported to the endoplasmic reticulum (ER), where esterification of free cholesterol by acyl-coenzyme A:cholesterol acyltransferase (ACAT) [27] takes place. Cholesterol esters formed by ACAT are stored as lipid droplets that are hydrolyzed by the cytoplasmic neutral cholesterol ester hydrolase. Tightly regulated cholesterol homeostatic mechanisms include *de novo* synthesis and esterification of cholesterol as well as LDL receptor synthesis [28], [29].

In NPC mutant cells, the endocytic uptake of LDL and the hydrolysis of LDL-derived cholesteryl esters to unesterified cholesterol in LE/LY are normal. However, the rate of cholesterol efflux from the LE/LY is severely impaired [30], [31], [32], resulting in altered regulation of cholesterol homeostasis [20], [23], [33]. Hence, despite the high levels of intracellular cholesterol, the rates of the synthesis of both cholesterol and LDL receptors are elevated, while the rate of cholesterol esterification by ACAT is diminished [20], [31], [32], [34]. Additionally, the amount of cholesterol transported from LE/LY to the plasma membrane is reduced [35]. LE/LY with high levels of cholesterol and other lipids such as bis-(monoacylglycerol)-phosphate contain multi-layered internal whorls of membrane. Cholesterol in the LSOs can be visualized by staining with filipin, a fluorescent polyene antibiotic [36], [37].

No fully effective treatment is available to date for NPC patients [38]. Based on the current knowledge of NPC disease, potential targets may mediate reduction of LE/LY lipid storage (cholesterol and/or glycosphingolipids). In a search for chemical compounds that could restore normal cholesterol distribution in NPC mutant cells, an automated microscopy screen was developed to identify compounds that partially correct cholesterol accumulation in Chinese hamster ovary (CHO) NPC1-deficient cultured cells [39]. This automated assay quantifies sterol accumulation in the LSOs based on images of the cells labeled with filipin. A good correlation was found between the amount of cholesterol in the cells based on the filipin staining and the amount of cellular cholesterol determined by gas chromatography (GC) [39]. From an initial screen of 14,956 combinatorially synthesized compounds, 14 compounds that reduced filipin staining of the LSOs at 10 µM were identified. From a follow-up screen of an additional 3,962 compounds, seven compounds that are effective at lower concentrations (123 nM to 3 µM) [39] were selected. Chemical structures of these commercially available, screen-selected chemicals were published previously [39].

In the work presented herein the mechanisms by which screen-selected sterol-lowering compounds reduce cholesterol levels in the LSOs in cultured NPC1 CT60 cells were examined. The mutant CT60 cells [40], with premature translational termination of the NPC1 protein, are derived from 25RA, a CHO cell line with a partial gain of function mutation in the SREBP (sterol regulatory element binding protein) cleavage-activating protein (SCAP) [41]. The partial SCAP mutation of the CT60 cells exacerbates the cholesterol-loading phenotype, a benefit in the analysis of cholesterol transport and metabolism in NPC1 cells. Here, the impact of the screen-selected compounds on cholesterol efflux to extracellular acceptors, the uptake of lipoprotein-derived cholesterol, and the amount of cholesteryl esters in compound-treated cells was studied. We report that for each compound the decrease in cholesterol level in the LSOs of the NPC1 mutant cultured cells could be explained by at least one of the following: increased efflux to extracellular acceptors, decreased uptake of lipoprotein-derived cholesterol, or increased levels of cholesteryl esters. Analyzing the general mechanisms of action through which the molecules reduce sterol storage in the cells could help identify molecular targets of the compounds. This approach has proven successful, since LAL was determined as the target of several of the screen-selected molecules [42]. We have included some of the previously published results on the LAL inhibitors [42] herein to give the reader the overall picture of the effects of the studied compounds on the cholesterol transport and metabolism as inferred from the biochemical analysis of the described pathways.

## Materials and Methods

### Materials

Chemical compounds were purchased from Chemical Diversity, Inc. (San Diego, CA). Their chemical structures are provided in the supplemental material (Figures S1 and S2) and also in [39]. Compound stocks of 10 mM in dimethyl sulfoxide (DMSO) were stored at −20°C.

Cell medium Ham's F12, fetal bovine serum (FBS), calf serum, Hank's balanced salt solution and Alexa Fluor 546 dye were all obtained from Invitrogen Corporation (Carlsbad, CA). [1,2-^3^H]-cholesterol (51.2 Ci/mmol) and [1-^14^C]-oleic acid (50 mCi/mmol) were purchased from PerkinElmer Life Science (Boston, MA). Solvents, bovine serum albumine (BSA) 99% fatty-acid, 4-(2-hydroxyethyl)-1-piperazine ethane sulphonic acid (HEPES), filipin, paraformaldehyde (PFA), mevinolin were all from Sigma Chemicals (St. Louis, MO). Sodium DL-mevalonate was prepared from DL-mevalonic acid lactone (Sigma Chemicals, MO) according to published procedure [43].

MetaMorph image analysis software was from MDS Analytical Technologies (Downington, PA).

### Preparation of lipoprotein-deficient serum (LPDS) and low-density lipoprotein (LDL)

LPDS was obtained from FBS adjusted to a final density of 1.2 g/ml [24]. LDL was obtained from human plasma, as described in [24]. LDL was labeled with 1,1′-dioctadecyl-3,3,3′,3′-tetramethylindocarbocyanine perchlorate (DiI) (Invitrogen Corporation, Carlsbad, CA) as described elsewhere [44], [45].

### Cell lines

NPC1 mutant cell line CT60 and the parental cell line 25RA were provided by Prof. T.Y. Chang (Dartmouth Medical School, Hanover, NH).

Cells were grown in growth medium [Ham's F12 medium, 1% Penicillin/Streptomycin, 2 g/L glucose, 1.176 g/L sodium bicarbonate] supplemented with 10% FBS. Growth medium containing 5.5% FBS and 20 mM HEPES was designated medium A.

### Evaluation of cholesteryl esters by GC

CT60 cells were plated in Falcon 12-well plates (VWR International, Inc., Bridgeport, NJ) in growth medium with 10% FBS, and after 24 h 10 µM of compound was added. The procedure described in [39] was followed. The amount of cholesteryl ester per µg protein was calculated for the solvent- and compound-containing wells.

### Incorporation of [^14^C]-oleic acid into cholesteryl esters

CT60 cells plated in Falcon 24-wells were grown for 16–18 h in medium A containing 10 µM chemical compound and 0.1 mM [^14^C]-oleate bound to albumin (prepared as described in [24]). The procedure described in [42] was followed. The number of pmoles of cholesteryl-[^14^C]-oleate per µg protein was calculated for each well.

### Cholesterol efflux

Cells grown in Falcon 24-well plates in growth medium with 10% FBS were labeled with 1 µCi/ml [^3^H]-cholesterol for 24 h. The procedure described in [46] was followed. Efflux was estimated as the radioactivity of the supernatant relative to the sum of the radioactivity of supernatant and cell monolayer.

### LDL uptake

Two different assays were used:

#### a. Short term uptake of DiI-LDL

CT60 cells were plated in Costar 96-well black polystyrene flat, clear bottomed tissue culture treated plates (Corning, Inc., NY) in growth medium supplemented with 10% FBS. After 24 h incubation at 37°C, medium was changed to one containing 5% LPDS. Following 16–18 h incubation at 37°C, the plate was washed with phosphate buffered saline (PBS) (pH 7.4) using a Bio-Tek Elx405 plate washer (Bio-Tek Intruments Inc., Winooski, VT). A solution of 10 µM compound and 30 µg/ml DiI-LDL prepared in 5% LPDS-medium was added to the cells. To the control samples, DMSO was added (0.1% v/v) instead of the compound. Cells were incubated for 35 min at 37°C and then washed with PBS, fixed with 1.8% PFA for 10 min, and then stained with filipin (50 µg/ml, 45 min). Images were acquired using Discovery-1 imaging system from Molecular Devices (MDS Analytical Technologies) equipped with a Xenon-arc lamp from Perkin Elmer (Waltham, MA), a 10 X Plan Fluor 0.3 NA objective from Nikon (Melville, NY) and a Photometrics CoolSnap HQ camera (1392×1040 pixels) from Roper Scientific (Tucson, AZ). For filipin, the following filter set was used: [360 nm (40 nm bandpass) excitation filter, 365 DCLP (DiChroic Long Pass) filter, 480 nm (40 nm bandpass) emission filter], whereas for DiI, the filter set was: [535 nm (40 nm bandpass) excitation filter, Chroma 51001 bs DiChroic filter, 610 nm (60 nm band pass) emission filter]. Images were acquired at four sites per well using 2×2 binning. All images were corrected for background and shading [39]. Cell area was determined based on the filipin images, and the average DiI intensity per cell area was measured.

#### b. Long term uptake of DiI-LDL

Cells plated in Costar 96-well plates were treated for 18 h with 10 µM compound and 6 µg/ml DiI-LDL in growth medium containing 10% FBA. The assay described in [39] was followed. The average DiI intensity per cell area was measured as described above.

### Alpha 2-macroglobulin (α2M) uptake

CT60 cells were grown on poly-D-lysine-treated coverslip dishes in growth medium with 10% FBS. After 48 h incubation at 37°C, cells received medium A containing 100 µg/ml of receptor-activated α2M [47], [48] conjugated with Alexa Fluor 546 (α2M-Alexa 546) and also 10 µM compound. After a pulse of 1 h at 37°C, cells were fixed with 1.8% PFA for 20 min at room temperature. Cells were then washed with PBS, and then free cholesterol was stained with filipin (50 µg/ml) for 90 min at room temperature. Wide-field fluorescence images were acquired using a Leica DMIRB microscope (Leica Mikroscopie und Systeme GmbH, Wetzlar, Germany) equipped with a Princeton Instruments cooled charged-coupled device camera (Princeton, NJ) driven by MetaMorph System software. Images were acquired with a high magnification oil immersion objective: 25x, 1.4 NA. The following filter set was used to image filipin: [360 nm (40 nm bandpass) excitation filter, 400 nm dichroic filter, and 470 nm (40 nm bandpass) emission filter]. To minimize photobleaching, a 1.5% neutral density filter was utilized. To image Alexa 546 Fluor a standard rhodamine filter set was used: [545 nm (30 nm bandpass) excitation filter, 570 nm longpass dichroic filter, and 610 nm (75 nm bandpass) emission filter]. Once images were corrected for background, cell area was determined based on the filipin images, and then the average Alexa-546 intensity per cell area was measured.

### Inhibition of *de novo* synthesis of cholesterol

CT60 cells were plated in Costar 96-well plates (Corning, Inc., NY) in growth medium supplemented with 10% FBS. After 24 h incubation at 37°C, cells were treated with medium A containing mevinolin (20 µM) and mevalonate (230 µM) [49]. For control cells only medium A was added. Following 18 h incubation at 37°C, cells were washed with PBS, fixed with 1.8% PFA for 10 min, and stained with filipin (50 µg/ml, 45 min). Images were acquired using Discovery-1 (see above), and the analysis was carried out using Metamorph Discovery-1 image-analysis software and custom-designed analysis assays called the “filipin intensity assay” and “LSO compartment ratio assay” which have been described in detail [39]. Briefly, a low threshold was set to define the area of the cells, and a high threshold was set to identify the filipin-stained LSO regions. Filipin intensity assay describes the total filipin intensity above the lower threshold divided by the number of pixels above the lower threshold. The LSO compartment ratio describes the total filipin intensity above the high threshold divided by the cell area (µm^2^) as defined by the low threshold. Based on these two assays, cholesterol content in the cell and in the LSOs, respectively was evaluated before and after treatment with mevinolin/mevalonate.

## Results

Cholesterol levels in the LSOs of NPC-deficient cells are very high due to impaired efflux of the sterol from these organelles [30], [31], [32]. With the use of a fluorescence automated microscopy screen, small molecules that decrease cholesterol in the LSOs of NPC1 mutant cultured cells were identified [39]. The cholesterol-lowering effect of the screen-selected chemicals was validated upon quantification by GC of the cellular cholesterol [39]. The structures of the chemicals are presented in Supplemental material (Figures S1 and S2). The majority of the chemicals did not exhibit cytotoxic effects on NPC1 mutant cells treated for 24 h with 10 µM compound [39]. Based on cytotoxicity inferred from assays measuring cell count and lactate dehydrogenase release [39], compound 2a12 was eliminated from further studies. In addition, compounds 1a2 and 2a3 were found to quench filipin fluorescence by more than 20%. Hence, 1a2 and 2a3 were also eliminated from further studies.

Here, the effect of the remaining 18 cholesterol-lowering, screen-selected chemicals on intracellular cholesterol pathways was studied. The effects of the compounds on cholesterol efflux from cells, cholesterol uptake *via* lipoprotein endocytosis, and the amount of cholesteryl esters in the cells were investigated. The compounds did not show a uniform profile in terms of their effects on these processes, indicating that their sterol-lowering effects are based on several different mechanisms. Each compound influenced one or more of these processes. Based on the analysis of the pathways affected by seven of these cholesterol-lowering small molecules (1a4, 1a11, 1a14, 2a8, 2a9, 2a13, 2a15), an in depth study revealed that these chemicals are inhibitors of LAL in CHO cells [42]. Moreover, we published data indicating that compound 1a13 increased esterification by ACAT and cholesterol efflux in both NPC mutant and normal cells [46]. Herein, we have retained the data on the LAL inhibitors (1a4, 1a11, 1a14, 2a8, 2a9, 2a13, 2a15) [42] and also on compound 1a13 [46] to give the reader an overall sense of all the processes affected by each compound since only the effect of these chemicals on specific processes was discussed in the previously published work. Recognizing the cellular mechanisms impacted by cholesterol-lowering chemicals could help reveal additional molecular target(s) of these molecules, a difficult task associated with any cell-based screening processes.

### Evaluation of cholesteryl esters by GC in compound-treated cells

**Figure 1:**
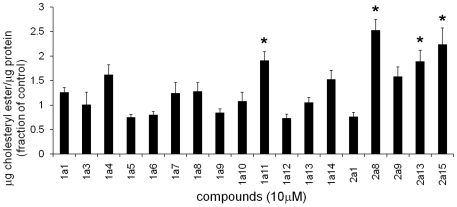
Quantification of cholesteryl ester levels by GC in CT60 cells treated with compounds. CT60 cells were treated with compound (10 µM) overnight at 37°C and cellular lipids were extracted with hexane/isopropyl alcohol. Total cholesterol (after saponification) and free cholesterol were measured, and the difference between these values was taken as the amount of cholesteryl ester. The amount of cholesteryl ester in the cell per µg cell protein is reported (all values were referenced to the solvent-treated cells, to pool data from separate experiments for statistical analysis purposes). For solvent-treated (control) CT60 cells, the mean value was 0.042±0.004 µg cholesteryl ester/µg cell protein. Experimental values for compound-treated cells were calculated as fraction of the control and are presented as mean ± S.E. (*p<0.01). Two to four independent experiments (6≤n≤12) were conducted. Data for compounds 1a4, 1a11, 1a14, 2a8, 2a9, 2a13 and 2a15 have been presented in [42].

The cholesteryl ester content of compound-treated and untreated cells was quantified by extraction of cellular lipids, saponification, and separation on a GC column (Figure 1). It can be seen that the effects of the compounds on cholesteryl ester content were variable. Seven compounds (1a4, 1a11, 1a14, 2a8, 2a9, 2a13 and 2a15) increased the cholesterol ester levels in the cells to at least 150% of the control value [42]. The remaining chemicals either did not affect the amount of cholesteryl ester significantly or decreased it.

### Incorporation of [^14^C]-oleic acid into cholesteryl esters

**Figure 2:**
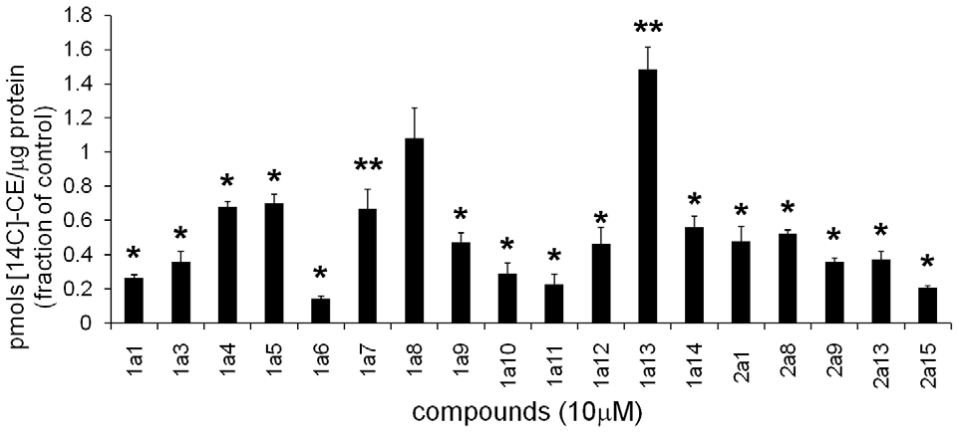
Incorporation of [^14^C]-oleic acid into cholesteryl-[^14^C] esters in CT60 cells treated with compounds. CT60 cells were plated in growth medium with 10% FBS, at 37°C. After 24 h, the medium was changed to medium A containing10 µM compound and 0.1 mM [^14^C]-oleate bound to albumin. After 16–18 h at 37°C, cellular lipids were extracted with organic solvents and separated by thin layer chromatography. Data analysis was performed using ImageQuant. The number of pmoles of cholesteryl-[^14^C]-oleate per µg cell protein was calculated. For solvent-treated (control) CT60 cells, the mean value was 14±1. Experimental data of compound-treated cells were calculated as fractions of the control (to pool data from separate experiments for statistical analysis purposes) and are presented as mean ± S.E. (*p<0.01 and **p<0.02). Three to five independent experiments (10≤n≤18) were conducted. Data for compounds 1a4, 1a11, 1a14, 2a8, 2a9, 2a13 and 2a15 have been presented in [42] and for 1a13 in [46]. [14C]-CE: cholesteryl-[^14^C]-ester.

To further examine the effect of the compounds on cholesterol esterification, the incorporation of [^14^C]-oleic acid into cholesteryl-[^14^C] esters in cells treated with the selected compounds under the screening conditions (18 hour incubation in FBS-containing medium) was investigated. This assay analyzes whether the compounds affect the transport of free cholesterol from the LSOs to ACAT in the ER. As shown in Figure 2, only compound 1a13 increased ACAT esterification significantly after 18 h treatment of the CT60 cells [46]. The increase in ACAT esterification shown by compound 1a8 was not statistically significant, and all the other 16 compounds caused a decrease of [^14^C]-oleic acid incorporation into esters as compared to the control. The effect of compounds on cholesterol esterification by ACAT was additionally tested by treating the cells with [^14^C]-cholesterol and quantifying [^14^C]-cholesteryl-oleate formed before and after addition of the chemical to the cells. Once again, only compound 1a13 caused an increase in the incorporation of cholesterol into esters, while all other chemicals decreased esterification (not shown).

The seven compounds (1a4, 1a11, 1a14, 2a8, 2a9, 2a13 and 2a15) that did increase cholesteryl esters levels in the cells (Figure 1 and [42]) also inhibited hydrolysis of cholesteryl esters in LE/LY [42]. However, these seven chemicals did not increase the ACAT esterification (Figure 2 and [42]). A published study identified these seven chemicals as novel inhibitors of LAL in the CHO cells [42].

The results described above show that except for the seven chemicals identified as novel inhibitors of LAL in NPC-deficient CT60 cells [42], and for compound 1a13 (which promotes ACAT-driven esterification), the remaining chemicals did not affect cholesterol esterification. Consequently, other cholesterol-related cellular mechanisms were investigated to reveal the mechanisms by which these molecules reduce cellular levels of cholesterol. Since each chemical might affect more than one mechanism, the studies described below included all 18 screen-selected chemicals.

### Cholesterol efflux

**Figure 3:**
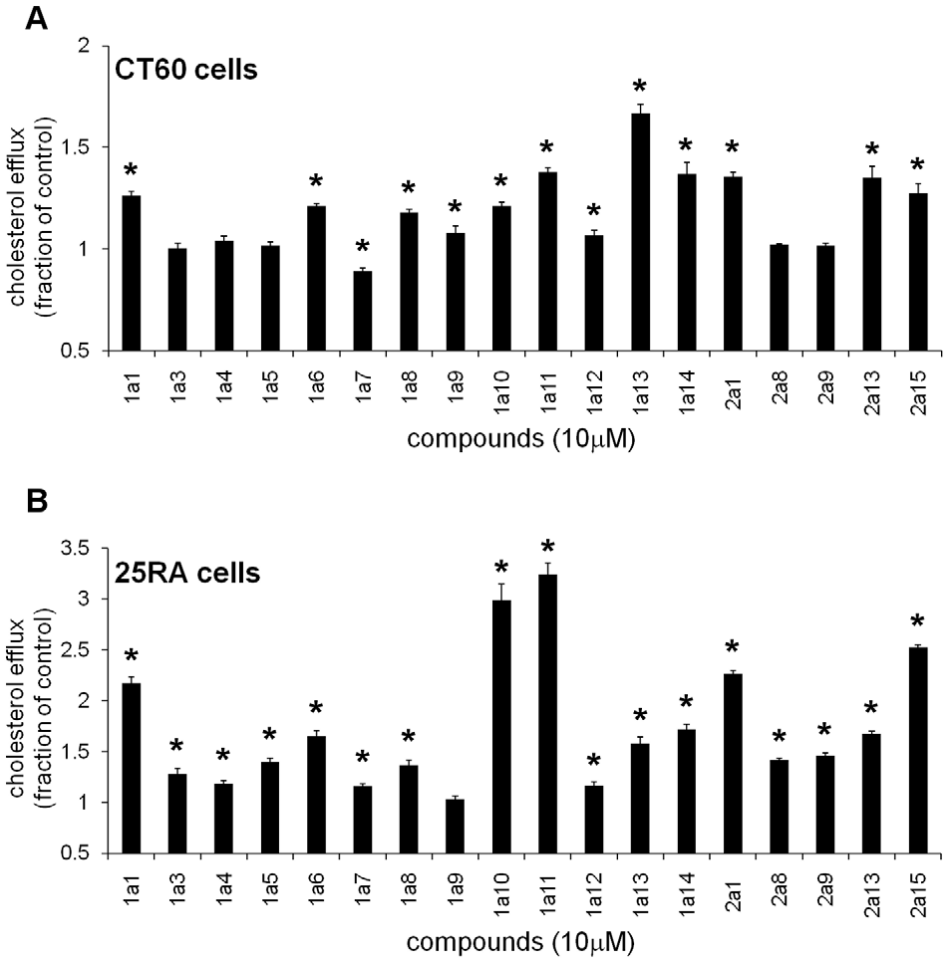
Cholesterol efflux in the presence of compounds. Cells (CT60 or 25RA) were grown for 24 h in growth medium with 10% FBS, at 37°C and then labeled with 1 µCi/ml [^3^H]-cholesterol for another 24 h at 37°C. Then cells were incubated for 2 h at 37°C in growth medium with 0.2% BSA. Medium was replaced with medium A containing 10 µM compound. After 16–18 h chase at 37°C, the medium and cell monolayer were collected. Efflux was expressed as the radioactivity of the supernatant relative to the sum of the radioactivity of supernatant and cell monolayer. The mean value of the efflux of the control (DMSO-treated) cells was (6.9±0.3)% and (6.6±0.4)% for CT60 and 25RA cells, respectively. Experimental data of compound-treated cells were calculated as fractions of the control (to pool data from separate experiments) and are presented as mean ± S.E. (*p<0.01). Two to six independent experiments (10≤n≤26) were conducted for CT60 cells, and two independent experiments (6≤n≤12) for 25RA cells. Data for compound 1a13 have been presented in [46].

The effect of the 18 compounds on cholesterol efflux to extracellular acceptors was studied by incubating NPC1 mutant CT60 cells with [^3^H]-cholesterol for 24 h, followed by a chase for 16–18 h in the presence of compound (Figure 3A). Nine compounds (1a1, 1a6, 1a10, 1a11, 1a13 [46], 1a14, 2a1, 2a13, 2a15) increased cholesterol efflux to at least 120% of the control value. One compound (1a7) decreased cholesterol efflux. The effect of the 18 compounds on cholesterol efflux was also investigated in the parental, non-NPC cell line, 25RA (Figure 3B). Interestingly, all nine compounds that increased efflux in NPC1 mutant CT60 cells also increased cholesterol efflux from parental 25RA cells (an increase to at least 150% of the control value was observed – Figure 3B). The remaining compounds affected the efflux in 25RA to a lesser extent or not at all. Based on these cholesterol efflux studies, these nine compounds (1a13 in particular [46]) could be considered valuable candidates for promoting cholesterol efflux to extracellular acceptors.

### LDL and α2M uptake

The effect of the compounds on cholesterol uptake *via* lipoprotein endocytosis was explored using DiI-LDL. A short-pulse assay (35 min incubation with DiI-LDL) was designed to test for chemicals' direct effects on LDL endocytosis, whereas a long-pulse assay (18 hours incubation with DiI-LDL) was a way to check for secondary effects such as down regulation of the expression of LDL receptor. The effect of 1a13 has been already published [46], and experimental results indicated no direct effect on LDL binding.

**Figure 4:**
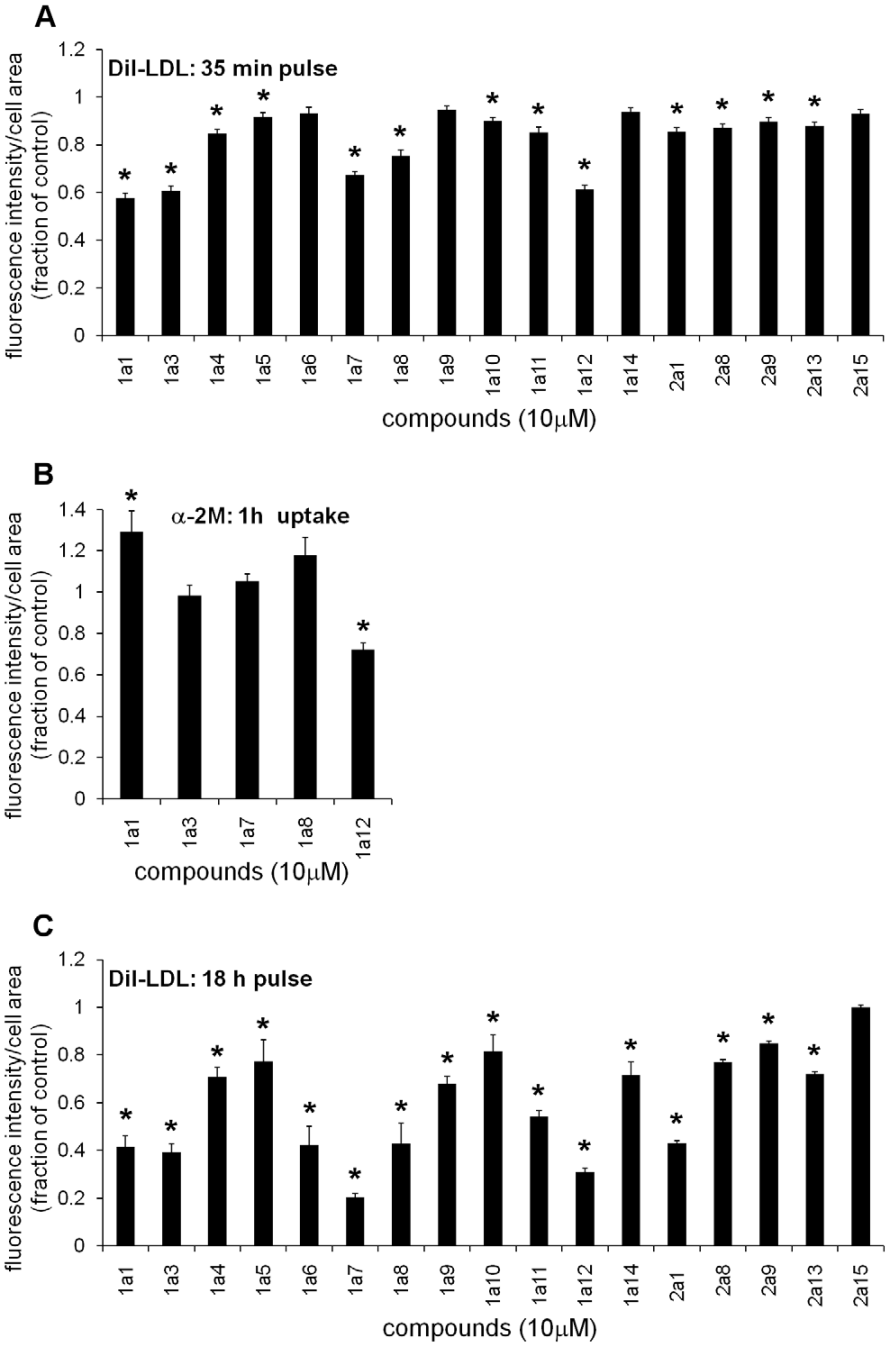
Internalization of DiI-LDL and α2M-Alexa 546 by CT60 cells during incubation in the presence of compounds. (**A**) CT60 cells were grown in growth medium with 10% FBS for 24 h at 37°C, and then the medium was changed to 5% LPDS. After 16–18 h, 10 µM compound and 30 µg/ml DiI-LDL were added. Cells were incubated for 35 min at 37°C, fixed and stained with filipin. The average DiI intensity per cell area was measured. Experimental data of compound-treated cells were calculated as fractions of the control (to pool data from separate experiments) and are presented as mean ± S.E. (*p<0.01). Four independent experiments (n = 12) were conducted. (**B**) CT60 cells were grown on glass coverslip bottom dishes. After 48 h incubation at 37°C, cells were incubated for 1 h in medium A containing 100 µg/ml of α2M-Alexa 546 and also 10 µM compound. Cells were then fixed and stained with filipin. The average Alexa-546 intensity per cell area was measured. Experimental data of compound-treated cells were calculated as fractions of the control and are presented as mean ± S.E. (*p<0.01). Two to four independent experiments (10≤n≤19) were conducted. (**C**) CT60 cells were plated in growth medium with 10% FBS, and after 24 h medium A with DiI-LDL (6 µg/ml) and 10 µM compound was added. After 18 h at 37°C cells were fixed and stained with filipin. The average DiI intensity per cell area was measured. Experimental data of compound-treated cells were calculated as fractions of the control and are presented as mean ± S.E. (*p<0.01). Three independent experiments (n = 9) were conducted. Experimental results for compounds 2a1, 2a8, 2a9, 2a13, 2a15 have been published previously [39]. Data for compound 1a13 have been obtained using the procedure described in [46] and experimental results indicated no effect on LDL binding.

In the short-pulse assay (Figure 4A), five compounds (1a1, 1a3, 1a7, 1a8, 1a12) caused a moderate to strong decrease of LDL uptake (the values corresponding to compound-treated samples were 50–80% of the control value). The rest of the compounds caused a smaller decrease in LDL uptake.

To check for general effects on endocytosis, the uptake of α2-macroglobulin (α2M), a serum protease inhibitor, was also examined for the five chemicals that caused the strongest effect on the short-term uptake of DiI-LDL. The uptake of α2M is mediated by the LDL receptor-related protein [47], [48], one of the seven members of the LDL receptor superfamily that is involved in a variety of biological processes, including lipoprotein metabolism, degradation of proteases and cellular entry of bacterial toxins and viruses. Compounds that induced the strongest inhibition of LDL uptake were tested for their effect on a 1 h uptake of α2M conjugated with Alexa Fluor 546 (α2M-Alexa 546) (Figure 4B). Compound 1a1 produced a small increase in α2M uptake, and 1a12 reduced α2M uptake, while the rest of the selected chemicals did not affect α2M uptake, suggesting that they do not perturb general endocytic processes in the cells.

The conditions for the 18 h LDL-uptake assay (Figure 4C) followed closely the screening procedure used for the selection of the compounds used in this study. It can be seen that all of the compounds, except 2a15, caused inhibition of LDL internalization during the 18 h treatment. Experimental results for the compounds 2a1, 2a8, 2a9, 2a13, 2a15 have been reported previously [39]. As indicated before, 1a13 also decreased uptake of LDL in an 18 hour assay [46]. The strong inhibition of LDL uptake upon long incubation of the cells with several compounds may reflect secondary effects such as down regulation of LDL receptor expression. CT60 cells have only a partial gain of function mutation in SCAP [40], which allows them to exhibit, at least to some degree, cholesterol-regulatory homeostatic mechanisms.

### Inhibition of the *de novo* synthesis of cholesterol

The effect of the inhibition of cholesterol biosynthesis on cholesterol levels in CT60 cells was studied under experimental conditions similar to those used for the screening assay. CT60 cells were incubated for 18 h in the presence of both mevinolin (20 µM) and mevalonate (230 µM) [49]. Since endogenous production of mevalonate from acetyl-CoA is inhibited by mevinolin, small amounts of mevalonate were added to the cell to allow production of isoprenoids vital for the cell growth [50]. The effect of mevinolin/mevalonate treatment on cholesterol level in the cells was estimated using the two filipin-based assays previously described [39]. In both assays, experimental results were normalized to the average value of control cells that did not receive mevinolin/mevalonate. Inhibition of cholesterol biosynthesis did not affect the overall cholesterol levels in the cells, as no effect was seen in the average filipin intensity assay (1.00±0.01). Furthermore, only a very small decrease of the cholesterol levels in the LSOs was observed upon mevinolin/mevalonate treatment, as indicated by the LSO filipin assay (0.96±0.01, p = 0.02).

## Discussion

Chemical compounds studied in this work have been shown to decrease overall cholesterol levels and cholesterol in the LSOs of NPC1-deficient CHO cells [39]. The goal of this study was to analyze which cholesterol transport processes and metabolic pathways were affected by the compounds, as a first step in identifying molecular targets of these compounds. Such information can help to determine which chemicals could be of interest in future investigations related to NPC or cholesterol metabolism in general. The decrease of cholesterol in compound-treated NPC1-deficient cells could be explained by one or more of the following: increased efflux to extracellular acceptors, decreased uptake of LDL-derived cholesterol, increased esterification of cholesterol or decreased hydrolysis of cholesteryl esters. The latter two would lead to increased levels of cholesteryl ester in cells. All these possibilities were taken into consideration, and the effect of each compound is summarized in Table 1. Experimental results showed that each high throughput screen-selected chemical affects at least one of the studied processes.

**Table 1:** Summary of the effects of screen-selected cholesterol-lowering compounds in CT60 cells.

Compound No.	Endocytosis of DiI-LDL. Short term uptake assay.	Efflux to extracellular acceptors.	Cholesteryl ester levels. GC assay.	The most important effect(s) of the chemicals, to explain cholesterol decrease in the LSOs of CT60 cells.
1a6	↓**	↑↑*	-	
1a9	↓**	↑*	-	
1a10	↓*	↑↑*	-	increased efflux
1a13	-	↑↑↑*	-	
2a1	↓*	↑↑*	-	
1a4	↓*	↑**	↑↑↑**	increased levels of
2a8	↓*	-	↑↑↑*	cholesteryl ester
2a9	↓*	-	↑↑↑**	
1a11	↓*	↑↑*	↑↑↑*	
1a14	↓**	↑↑*	↑↑↑**	increased efflux and
2a13	↓*	↑↑*	↑↑↑*	increased ester levels
2a15	↓**	↑↑*	↑↑↑*	
1a3	↓↓*	-	-	
1a5	↓*	-	-	
1a7	↓↓*	↓*	-	decreased LDL uptake
1a8	↓↓*	↑*	-	
1a12	↓↓*	↑*	-	
1a1	↓↓*	↑↑*	↑↑**	increased efflux and ester levels, and decreased LDL uptake

Compounds were grouped based on their effect on key steps in cholesterol transport and metabolism that explain the decrease of the sterol in the LSOs in CT60 cells. A compound is considered to affect a process if its effect meets the criterion of statistical relevance according to the student's t-test;

*p<0.01 and

**p<0.1. Compound-triggered increased effect with respect to the control is indicated with an upward arrow (↑), while a decrease is shown with a downward arrow (↓). To distinguish between several compounds that affect the same process, a second criterion was used. A weak increase is considered one that induces a change that is less than 20% and is indicated in the table by one arrow. A moderate change (20–50%) is shown by two arrows, whereas a strong increase corresponds to a change of more than 50% and is shown with three arrows.

Compounds could act directly by promoting efflux from LSOs or indirectly by reducing cholesterol levels elsewhere in the cell. For example, inhibition of the uptake of LDL-derived cholesterol and/or the decrease in the hydrolysis of cholesteryl esters could lead to a reduction in the amount of free cholesterol produced in the LE/LY. Since in the NPC-deficient cells the efflux of cholesterol from the LE/LY is drastically diminished, a decline in the rate of cholesterol production in these organelles might be beneficial since there is evidence that sterol in the LSOs does slowly exchange with other cellular sterol pools [51], [52].

The selective inhibition of LDL uptake as compared to uptake of α2M by some compounds suggests that they may be relatively selective inhibitors of LDL uptake. After binding to the LDL receptor-related protein, α2M enters cells by clathrin-dependent receptor-mediated endocytosis and is delivered to lysosomes [53]. The internalized receptors (both the LDL receptor-related protein and the LDL receptor) are recycled back to the cell surface. Although the overall mechanisms of uptake of LDL receptor-related protein and LDL receptor are very similar, some differences have been reported. Interestingly, knockout of autosomal recessive hypercholesterolemia, a clathrin adaptor protein that interacts with both receptors, greatly decreases LDL uptake with no effect on uptake of α2M by the LDL receptor-related protein [54]. Further work would be required to determine if interaction with autosomal recessive hypercholesterolemia protein is related to the selective effects of some compounds on LDL uptake.

Quantification by GC of the levels of cholesteryl ester in compound-treated cells indicated that seven chemicals (1a4, 1a11, 1a14, 2a8, 2a9, 2a13 and 2a15) increased ester levels in the NPC1-deficient CHO cells (Figure 1 and [42]). However, none of these chemicals increased ACAT-driven esterification (Figure 2 and [42]). An in depth study of the effects of these seven compounds revealed that they inhibit LAL, the lysosomal enzyme that hydrolyzes LDL-derived triacylglycerol and cholesteryl esters [42].

The assays used in this paper did not directly measure efflux of sterol from the LSOs. However, it is expected that sterol released from the LSOs would be available for either esterification by ACAT, which resides in the ER, or efflux to extracellular acceptors via the plasma membrane. Compound 1a13 was the only compound that increased cholesterol efflux *and* increased esterification by ACAT [46]. Moreover, in NPC1-deficient cells treated with 1a13, the total cellular cholesterol was increased slightly even as the filipin-labeled cholesterol in the LE/LY was decreased [39]. All these results indicate that cholesterol delivered via lipoproteins in the serum exits the LSOs upon treatment of the NPC1 mutant cells with 1a13, and the sterol becomes available for efflux to extracellular acceptors or esterification in the ER. These experimental observations make 1a13 a candidate for directly promoting sterol efflux from LSOs in NPC1-deficient and other cells.

We also evaluated the possibility of decreasing cholesterol levels in the cells through the inhibition of *de novo* synthesis of cholesterol. CT60 cells were treated with mevalonin, which is an inhibitor of hydroxymethylglutaryl CoA reductase (HMG-CoA reductase), the rate limiting enzyme in the biosynthetic pathway of cholesterol. As mentioned in the Results section, mevalonin triggered only a very small decrease in the sterol levels in the LSOs, as determined by the filipin-based assay. Therefore, even if some of the chemicals might have had inhibited HMG-CoA reductase and thereby decreased *de novo* cholesterol synthesis, this metabolic process is unlikely to be a key path to explain chemicals' ability to lower cholesterol accumulation in the LSOs in CT60 cells. Consistent with this result, attempts to alleviate NPC symptoms through the blockage of the endogeneous cholesterol synthesis have not been successful [55].

Much remains to be unraveled from further studies of the 18 cholesterol-lowering compounds that partially revert the NPC phenotype, as the molecular targets of only seven of the chemicals have been identified [42]. Experimental results summarized in Table 1 indicate that several compounds affect cholesterol efflux. The active mechanism of the cholesterol efflux to extracellular acceptors occurs via ATP binding cassette transporter A1 (ABCA1) and ATP binding cassette transporter G1 (ABCG1) [56]. ABCA1 promotes efflux of cholesterol and phospholipids to lipid-poor apolipoprotein A-1 or apolipoprotein E, while ABCG1 promotes efflux of cholesterol to high-density lipoprotein particles. However, the details of the mechanisms underlying the ABCA1- and ABCG1-mediated lipid efflux remain unclear. Nine chemicals promoted cholesterol efflux in both non-NPC and NPC1-deficient cells (1a1, 1a6, 1a10, 1a11, 1a13 [46], 1a14, 2a1, 2a13, 2a15). In general the effect on efflux, relative to the control, in 25RA cells was greater than in CT60 cells. One may correlate this experimental observation to the fact that in CT60 cells there is an impaired efflux of cholesterol from the LE/LY, compared to 25RA cells, so less cholesterol is available for transport to the plasma membrane and extracellular acceptors. Among the nine compounds indicated above, 1a10 and 1a11 induced the strongest effect on efflux in 25RA cells. For each of these two chemicals the relative increase in efflux in 25RA cells was over twice the level of the effect seen in CT60 cells. Future studies may reveal whether the impact on cholesterol efflux is a consequence of a direct effect on the ABC transporters.

Alternatively, one may consider the possibility that the effects of the chemicals on cholesterol efflux and/or the overall decrease of the sterol in the cell are results of the activation of the nuclear liver X receptor/retinoid X receptor (LXR/RXR). Oxysterols, intermediates or end products in the metabolic pathway of cholesterol, are endogenous LXR agonists and promote the induction of an array of key proteins, among which are the efflux receptors ABCA1 and ABCG1 [1], [57]. Studies have shown that in NPC mutant cells there is a defective ABCA1-dependent efflux [58] and a diminished production of endogeneous oxysterol ligands for LXR [59]. Published studies indicated that treatment with the LXR agonist T0901317 of *Npc1^−/−^* mice prolonged their life span, increased cholesterol excretion out of brain and improved slightly the neurological function [60]. Future analysis may indicate if any of the 18 sterol-lowering chemicals described herein (which includes the nine compounds that affect cholesterol efflux) are synthetic agonists of LXR or promote the formation of natural LXR ligands.

Knowing the cholesterol pathways affected by the screen-selected chemicals may help find relationships between chemicals' structures and their molecular targets. For instance, compound 1a13, which promotes efflux effectively, is structurally-related to 1a5, 1a6, 1a7 and 1a8. These compounds (except 1a7) increase efflux in non-NPC 25RA cells by at least 20%. It is interesting to note that 1a13 has a carboxyl group in the molecule, a group not present in the other compounds.

The results presented in this manuscript emphasize advantages of the biochemical analysis of the effects of screen-selected compounds on key transport and metabolic processes as a crucial step in the identification of molecular targets of the chemicals. Since the specific pathway(s) related to LDL receptor-mediated endocytosis affected by each compound have been identified, the selection of compounds for further studies may be prioritized. Synthesis of additional compounds, structurally derived from those presented in this work, could lead to the discovery of chemically more potent compounds, bearing structural features specific to their *in vivo* binding partners.

## Supporting Information

Figure S1 Chemical structures of the 14 cholesterol-lowering chemicals screen-selected from a first library of compounds.
(4.97 MB TIF)

Figure S2 Chemical structures of the seven cholesterol-lowering chemicals screen-selected from a second library of compounds.
(4.14 MB TIF)

## References

[1] Maxfield FR, Tabas I (2005). Role of cholesterol and lipid organization in disease.. Nature.

[2] Mukherjee S, Maxfield FR (2004). Lipid and cholesterol trafficking in NPC.. Biochim Biophys Acta.

[3] Chang TY, Reid PC, Sugii S, Ohgami N, Cruz JC (2005). Niemann-Pick type C disease and intracellular cholesterol trafficking.. J Biol Chem.

[4] Ikonen E, Holtta-Vuori M (2004). Cellular pathology of Niemann-Pick type C disease.. Semin Cell Dev Biol.

[5] Pagano RE (2003). Endocytic trafficking of glycosphingolipids in sphingolipid storage diseases.. Philos Trans R Soc Lond B Biol Sci.

[6] Sturley SL, Patterson MC, Balch W, Liscum L (2004). The pathophysiology and mechanisms of NP-C disease.. Biochim Biophys Acta.

[7] Vanier MT, Millat G (2003). Niemann-Pick disease type C.. Clin Genet.

[8] Carstea ED, Morris JA, Coleman KG, Loftus SK, Zhang D (1997). Niemann-Pick C1 disease gene: homology to mediators of cholesterol homeostasis.. Science.

[9] Naureckiene S, Sleat DE, Lackland H, Fensom A, Vanier MT (2000). Identification of HE1 as the second gene of Niemann-Pick C disease.. Science.

[10] Neufeld EB, Wastney M, Patel S, Suresh S, Cooney AM (1999). The Niemann-Pick C1 protein resides in a vesicular compartment linked to retrograde transport of multiple lysosomal cargo.. J Biol Chem.

[11] Patel SC, Suresh S, Kumar U, Hu CY, Cooney A (1999). Localization of Niemann-Pick C1 protein in astrocytes: implications for neuronal degeneration in Niemann- Pick type C disease.. Proc Natl Acad Sci U S A.

[12] Higgins ME, Davies JP, Chen FW, Ioannou YA (1999). Niemann-Pick C1 is a late endosome-resident protein that transiently associates with lysosomes and the trans-Golgi network.. Mol Genet Metab.

[13] Okamura N, Kiuchi S, Tamba M, Kashima T, Hiramoto S (1999). A porcine homolog of the major secretory protein of human epididymis, HE1, specifically binds cholesterol.. Biochim Biophys Acta.

[14] Ioannou YA (2005). Guilty until proven innocent: the case of NPC1 and cholesterol.. Trends Biochem Sci.

[15] Liscum L, Sturley SL (2004). Intracellular trafficking of Niemann-Pick C proteins 1 and 2: obligate components of subcellular lipid transport.. Biochim Biophys Acta.

[16] Scott C, Ioannou YA (2004). The NPC1 protein: structure implies function.. Biochim Biophys Acta.

[17] Zhang M, Sun M, Dwyer NK, Comly ME, Patel SC (2003). Differential trafficking of the Niemann-Pick C1 and 2 proteins highlights distinct roles in late endocytic lipid trafficking.. Acta Paediatr.

[18] Blanchette-Mackie EJ (2000). Intracellular cholesterol trafficking: role of the NPC1 protein.. Biochim Biophys Acta.

[19] Ohgami N, Ko DC, Thomas M, Scott MP, Chang CC (2004). Binding between the Niemann-Pick C1 protein and a photoactivatable cholesterol analog requires a functional sterol-sensing domain.. Proc Natl Acad Sci U S A.

[20] Pentchev PG, Comly ME, Kruth HS, Vanier MT, Wenger DA (1985). A defect in cholesterol esterification in Niemann-Pick disease (type C) patients.. Proc Natl Acad Sci U S A.

[21] Cruz JC, Sugii S, Yu C, Chang TY (2000). Role of Niemann-Pick type C1 protein in intracellular trafficking of low density lipoprotein-derived cholesterol.. J Biol Chem.

[22] Morris JA, Carstea ED (1998). Niemann-Pick C disease: cholesterol handling gone awry.. Mol Med Today.

[23] Liscum L, Faust JR (1987). Low density lipoprotein (LDL)-mediated suppression of cholesterol synthesis and LDL uptake is defective in Niemann-Pick type C fibroblasts.. J Biol Chem.

[24] Goldstein JL, Basu SK, Brown MS (1983). Receptor-mediated endocytosis of low-density lipoprotein in cultured cells.. Methods Enzymol.

[25] Goldstein JL, Dana SE, Faust JR, Beaudet AL, Brown MS (1975). Role of lysosomal acid lipase in the metabolism of plasma low density lipoprotein. Observations in cultured fibroblasts from a patient with cholesteryl ester storage disease.. J Biol Chem.

[26] Tall AR (2003). Role of ABCA1 in cellular cholesterol efflux and reverse cholesterol transport.. Arterioscler Thromb Vasc Biol.

[27] Chang TY, Chang CC, Cheng D (1997). Acyl-coenzyme A:cholesterol acyltransferase.. Annu Rev Biochem.

[28] Goldstein JL, DeBose-Boyd RA, Brown MS (2006). Protein sensors for membrane sterols.. Cell.

[29] Brown MS, Goldstein JL (1986). A receptor-mediated pathway for cholesterol homeostasis.. Science.

[30] Neufeld EB, Cooney AM, Pitha J, Dawidowicz EA, Dwyer NK (1996). Intracellular trafficking of cholesterol monitored with a cyclodextrin.. J Biol Chem.

[31] Liscum L, Ruggiero RM, Faust JR (1989). The intracellular transport of low density lipoprotein-derived cholesterol is defective in Niemann-Pick type C fibroblasts.. J Cell Biol.

[32] Sokol J, Blanchette-Mackie J, Kruth HS, Dwyer NK, Amende LM (1988). Type C Niemann-Pick disease. Lysosomal accumulation and defective intracellular mobilization of low density lipoprotein cholesterol.. J Biol Chem.

[33] Lin S, Lu X, Chang CC, Chang TY (2003). Human acyl-coenzyme A:cholesterol acyltransferase expressed in chinese hamster ovary cells: membrane topology and active site location.. Mol Biol Cell.

[34] Pentchev PG, Comly ME, Kruth HS, Tokoro T, Butler J (1987). Group C Niemann-Pick disease: faulty regulation of low-density lipoprotein uptake and cholesterol storage in cultured fibroblasts.. FASEB J.

[35] Wojtanik KM, Liscum L (2003). The transport of low density lipoprotein-derived cholesterol to the plasma membrane is defective in NPC1 cells.. J Biol Chem.

[36] Lefevre M (1988). Localization of lipoprotein unesterified cholesterol in nondenaturing gradient gels with filipin.. J Lipid Res.

[37] Castanho MA, Coutinho A, Prieto MJ (1992). Absorption and fluorescence spectra of polyene antibiotics in the presence of cholesterol.. J Biol Chem.

[38] Patterson MC, Platt F (2004). Therapy of Niemann-Pick disease, type C.. Biochim Biophys Acta.

[39] Pipalia NH, Huang A, Ralph H, Rujoi M, Maxfield FR (2006). Automated microscopy screening for compounds that partially revert cholesterol accumulation in Niemann-Pick C cells.. J Lipid Res.

[40] Cadigan KM, Spillane DM, Chang TY (1990). Isolation and characterization of Chinese hamster ovary cell mutants defective in intracellular low density lipoprotein-cholesterol trafficking.. J Cell Biol.

[41] Chang TY, Limanek JS (1980). Regulation of cytosolic acetoacetyl coenzyme A thiolase, 3-hydroxy-3-methylglutaryl coenzyme A synthase, 3-hydroxy-3-methylglutaryl coenzyme A reductase, and mevalonate kinase by low density lipoprotein and by 25-hydroxycholesterol in Chinese hamster ovary cells.. J Biol Chem.

[42] Rosenbaum AI, Rujoi M, Huang AY, Du H, Grabowski GA (2009). Chemical screen to reduce sterol accumulation in Niemann-Pick C disease cells identifies novel lysosomal acid lipase inhibitors.. Biochim Biophys Acta.

[43] Brown MS, Faust JR, Goldstein JL, Kaneko I, Endo A (1978). Induction of 3-hydroxy-3-methylglutaryl coenzyme A reductase activity in human fibroblasts incubated with compactin (ML-236B), a competitive inhibitor of the reductase.. J Biol Chem.

[44] Dunn KW, Maxfield FR (1992). Delivery of ligands from sorting endosomes to late endosomes occurs by maturation of sorting endosomes.. J Cell Biol.

[45] Pitas RE, Innerarity TL, Weinstein JN, Mahley RW (1981). Acetoacetylated lipoproteins used to distinguish fibroblasts from macrophages in vitro by fluorescence microscopy.. Arteriosclerosis.

[46] Cosner CC, Markiewicz JT, Bourbon P, Mariani CJ, Wiest O (2009). Investigation of N-aryl-3-alkylidenepyrrolinones as potential Niemann-Pick type C disease therapeutics.. J Med Chem.

[47] Strickland DK, Ashcom JD, Williams S, Burgess WH, Migliorini M (1990). Sequence identity between the alpha 2-macroglobulin receptor and low density lipoprotein receptor-related protein suggests that this molecule is a multifunctional receptor.. J Biol Chem.

[48] Kristensen T, Moestrup SK, Gliemann J, Bendtsen L, Sand O (1990). Evidence that the newly cloned low-density-lipoprotein receptor related protein (LRP) is the alpha 2-macroglobulin receptor.. FEBS Lett.

[49] Spillane DM, Reagan JW, Kennedy NJ, Schneider DL, Chang TY (1995). Translocation of both lysosomal LDL-derived cholesterol and plasma membrane cholesterol to the endoplasmic reticulum for esterification may require common cellular factors involved in cholesterol egress from the acidic compartments (lysosomes/endosomes).. Biochim Biophys Acta.

[50] Goldstein JL, Brown MS (1990). Regulation of the mevalonate pathway.. Nature.

[51] Pipalia NH, Hao M, Mukherjee S, Maxfield FR (2007). Sterol, protein and lipid trafficking in Chinese hamster ovary cells with Niemann-Pick type C1 defect.. Traffic.

[52] Dahl NK, Reed KL, Daunais MA, Faust JR, Liscum L (1992). Isolation and characterization of Chinese hamster ovary cells defective in the intracellular metabolism of low density lipoprotein-derived cholesterol.. J Biol Chem.

[53] Willingham MC, Maxfield FR, Pastan IH (1979). alpha 2 Macroglobulin binding to the plasma membrane of cultured fibroblasts. Diffuse binding followed by clustering in coated regions.. J Cell Biol.

[54] Jones C, Hammer RE, Li WP, Cohen JC, Hobbs HH (2003). Normal sorting but defective endocytosis of the low density lipoprotein receptor in mice with autosomal recessive hypercholesterolemia.. J Biol Chem.

[55] Reid PC, Lin S, Vanier MT, Ohno-Iwashita Y, Harwood HJ (2008). Partial blockage of sterol biosynthesis with a squalene synthase inhibitor in early postnatal Niemann-Pick type C npcnih null mice brains reduces neuronal cholesterol accumulation, abrogates astrogliosis, but may inhibit myelin maturation.. J Neurosci Methods.

[56] Yvan-Charvet L, Wang N, Tall AR (2010). Role of HDL, ABCA1, and ABCG1 transporters in cholesterol efflux and immune responses.. Arterioscler Thromb Vasc Biol.

[57] Beaven SW, Tontonoz P (2006). Nuclear receptors in lipid metabolism: targeting the heart of dyslipidemia.. Annu Rev Med.

[58] Choi HY, Karten B, Chan T, Vance JE, Greer WL (2003). Impaired ABCA1-dependent lipid efflux and hypoalphalipoproteinemia in human Niemann-Pick type C disease.. J Biol Chem.

[59] Frolov A, Zielinski SE, Crowley JR, Dudley-Rucker N, Schaffer JE (2003). NPC1 and NPC2 regulate cellular cholesterol homeostasis through generation of low density lipoprotein cholesterol-derived oxysterols.. J Biol Chem.

[60] Repa JJ, Li H, Frank-Cannon TC, Valasek MA, Turley SD (2007). Liver X receptor activation enhances cholesterol loss from the brain, decreases neuroinflammation, and increases survival of the NPC1 mouse.. J Neurosci.

